# Retraction: ‘RNA editing of microRNA prevents RNA-induced silencing complex recognition of target mRNA’ (2015), by Cui *et al.*

**DOI:** 10.1098/rsob.230403

**Published:** 2023-11-03

**Authors:** Open Biology editorial team

**Keywords:** RNA editing, miRNA-induced silencing complex, virus latency, adenosine deaminase acting on RNA

*Open Biol.* **5**, 150126 (Published online 1 December 2015). (https://doi.org/10.1098/rsob.150126)

Following an investigation, we have found duplicates in the figures presented (notably fig. 5. ‘The mechanism of viral miRNA editing in virus infection’ panels (*c,d*)). This paper has been identified among of a set of papers that present significant text and data duplication and therefore the data reported in this article are unreliable. Subsequent to this retraction notice, the authors contacted us to supply the original data, which confirmed the data substitution. After careful consideration, the Editorial Board decided that the retraction should remain in place. The authors disagree with the retraction.

The image integrity standards and policies for the journal can be found here: https://royalsocietypublishing.org/doi/10.1098/rsob.200165.

Jonathon Pines FRS

Editor-in-Chief, *Open Biology*.

